# Case report: Clinical and single-cell transcriptome sequencing analysis of a mixed gangliocytoma-adenoma presenting as acromegaly

**DOI:** 10.3389/fonc.2022.1088803

**Published:** 2022-12-08

**Authors:** Chao Li, Daqin Feng, Dabiao Zhou

**Affiliations:** ^1^ Department of Neurosurgery, The First Affiliated Hospital, Guangxi Medical University, Nanning, China; ^2^ Department of Neurosurgery, Beijing Tiantan Hospital, Capital Medical University, Beijing, China

**Keywords:** mixed gangliocytoma-adenoma, pituitary tumors, acromegaly, single-cell transcriptome sequencing, case report

## Abstract

**Background:**

Mixed gangliocytoma-adenoma (MGA) is a rare tumor of pituitary gland. It’s difficult to distinguish it from pituitary adenoma by clinical manifestations, imaging features or serological testing. Thus, the histopathological examination is still the golden standard for diagnosis. Besides, studies on molecular level are still lacking.

**Case information:**

In this case report, we described a 28-year-old male with MGA presenting as acromegaly, who suffered staging operation and post-operation gamma knife radiosurgery, but finally died of secondary hyperglycemic hyperosmolar collapse. A complete data including clinical, histopathological, ultrastructural and single-cell transcriptome level information were collected and analyzed.

**Conclusion:**

This case report detailed the only clinical and molecular report of MGA following operation and radiotherapy. Complete clinical data enhanced the understanding of the diagnosis and treatment of this disease. Besides, the single-cell transcriptome sequencing analysis further disclosed the intra-tumoral heterogeneity and provided support for subsequent basic research.

## Introduction

Mixed gangliocytoma-adenoma (MGA) is a kind of collision tumor of the pituitary gland, which was first written into World Health Organization (WHO) Classification of pituitary tumors in 2017 (1, 2). It is rarely seen among pituitary tumors, accounting for 0.29% approximately and often presenting the same kind of clinical symptoms as pituitary adenomas (3). Up to now, no more than 200 cases had been reported worldwide, and research on the diagnostic, therapeutic and molecular pathological aspects of the disease is still lacking (4). The aim of this study is to report a MGA, to be more precise, a growth hormone (GH) secreting type presenting as acromegaly. The clinical course, imaging features, blood hormone levels, histopathologic features, ultrastructural features and single-cell transcriptome characterization of the case were included in this study. This case report followed the CARE Guidelines (5).

## Case description

### Clinical course

**Figure 1:**
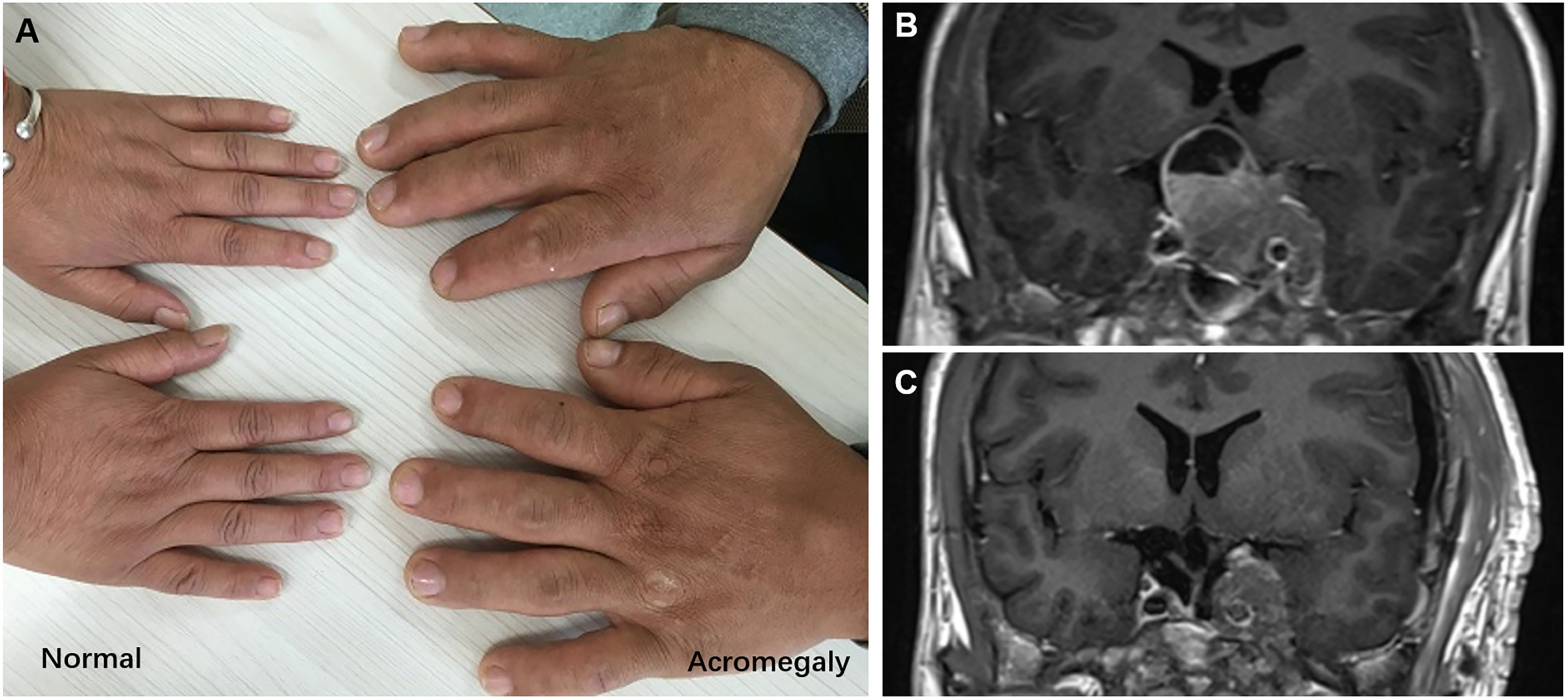
**(A)** The hands of normal and acromegaly people. **(B)** Preoperative MRI, 44mm× 27mm×37mm in size, with left cavernous sinus involvement, and internal carotid artery encased. **(C)** Postoperative MRI, show the residual tumor encircling the left internal carotid artery.

The patient was a 20-year-old Chinese male who developed symptoms and signs of acromegaly within three years prior to hospitalization, including enlargement of the hands, feet, nose and jaw, thicker skin, deepening of the voice, and myocardial hypertrophy, etc. **(** **Figure 1A** **)**. Over the one-month period before admition, he developed headache, hypertension, diabetes, reduced vision, visual field defects and polyuria. Blood hormone level screening showed the serum growth hormone level of him was over 40ng/mL (0.06-5.00ng/mL), and the Insulin-like growth factor-1(IGF-1) was 1567ng/mL(127~424ng/mL). The MRI revealed a solid-cystic lesion located in the enlarged and sunken sellar turcica, measuring about 44mm*27mm*37mm. The lesion presented with equal T1 and T2 signals, and showed significantly inhomogeneous enhancement. The MRI also showed an invasive behavior with mass effect on the optic chiasm, left cavernous sinus involvement, and internal carotid artery encased **(** **Figure 1B** **)**.

Due to the relatively large size of the tumor and the location both within the sellar and supra-sellar, it was difficult to remove it at once. So, the doctor suggested a staged tumor resection plan. For the first time, the tumor was removed from the sellar using a transnasal-sphenoidal approach. Intraoperatively, the tumor was seen to be grayish-red in color, with a heterogeneous texture and a relatively rich blood supply. The operation was successful with no postoperative complications like cerebrospinal fluid leakage. After the first surgery, the serum GH level decreased to some extent but was not well controlled and remained at about 18ng/mL (0.06-5.00ng/mL). Eleven months later, the patient underwent a second surgery with the frontolateral approach to remove the suprasellar and parasellar part of the tumor. However, due to the hard texture of the tumor encircling the left internal carotid artery, some of the tumor body remained **(** **Figure 1C** **)**, and the postoperative serum GH level still did not drop to normal level which was about 7ng/mL (0.06-5.00ng/mL). Three months after surgery, the patient further underwent gamma knife radiation therapy. Unluckily, it did not show significant efficacy after treatment. Then, the doctor suggested a growth inhibitor analog injection therapy, but the patients denied due to the weak economic condition. Four months later, the patient developed a severe hyperosmolar hyperglycemic syndrome with blood glucose exceeding 35 mmol/L, resulting in electrolyte disturbances and secondary coma. Finally, the patient died of ineffective resuscitation in 33 months after the first surgery. Time line of the clinical course was shown in **Figure S1**.

### Histopathological examination

**Figure 2:**
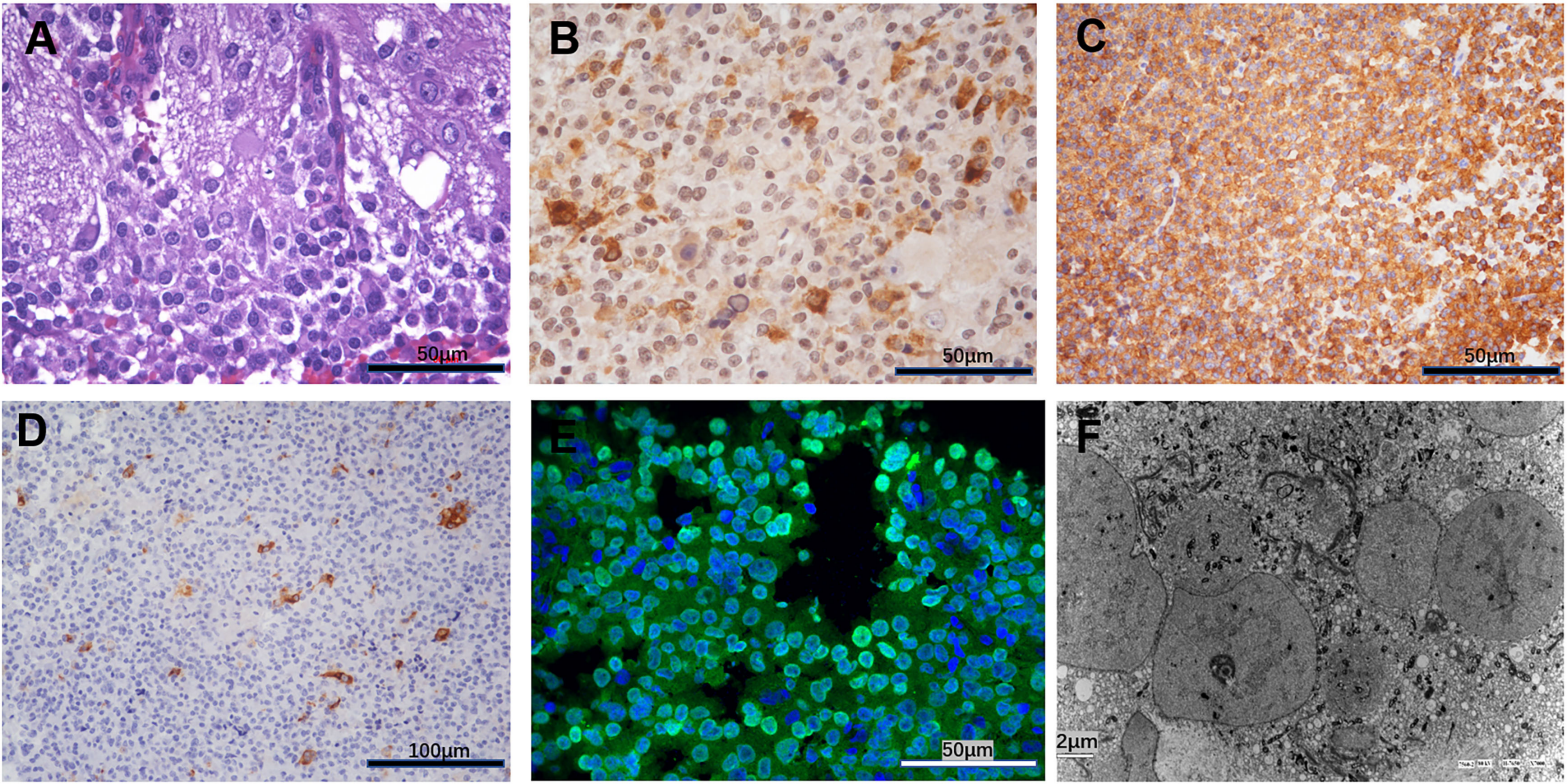
**(A)** Hematoxylin-eosin HE revealed cap-like gangliocytoma cells surrounding the adenoma cells. **(B)** Immunohistochemistry showed strongly positive for growth hormone. **(C)** Immunohistochemistry showed strongly positive for synapsin. **(D)** Immunohistochemistry showed scattered positive for Prolactin (PRL). **(E)** Immunofluorescent staining show strong positive for Pituitary Transcript Factor 1(Pit-1). **(F)** The ultrastructure observed by Electron microscope.

The hematoxylin-eosin (HE) staining revealed cap-like gangliocytoma cells surrounding the adenoma cells **(** **Figure 2A** **)**. Further immunohistochemistry profile showed strongly positive for GH and Synapsin, scattered positive for Prolactin(PRL) and Ck8/18, and about 5% monoclonal antibody (MIB)-1 proliferative index positivity **(** **Figures 2B** **–D** **)**. Immunofluorescent staining show strong positive for Pituitary transcript factor 1(Pit-1) **(** **Figure 2E** **)**, which further proved its Pit-1 lineage origin. These findings are characteristic of the diagnosis of MGA. Further, the ultrastructure observed by electron microscope showed that tumor cells were densely distributed, with nuclei of different sizes, intracytoplasmic fibrous vesicles, round secretory granules within the fibrous vesicles, and scattered capillaries in the interstitium **(** **Figure 2F** **)**. The suspicious gangliocytoma cells were large and contain abundant cytoplasm and Nissl granules.

### Single cell transcriptome sequencing analysis

**Figure 3:**
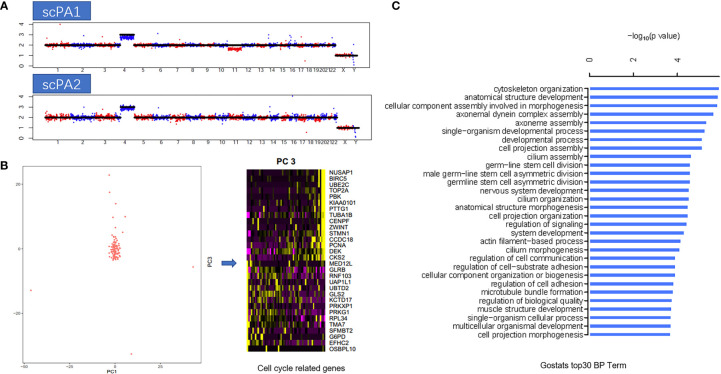
**(A)** Copy number variation (CNV) profiles of the two specimens: first surgery(scPA1) and second surgery(scPA2), show gain in chromosome 4 (both), and chromosome 11 deletion in scPA1 only, indicated the possible existence of temporal and spatial heterogeneity within the tumor. **(B)** Principal component analysis (PCA) showed that the cells were almost clustered together, and only little part of cells (7/209) had cell cycle heterogeneity (PC2 represented cell cycle related genes). **(C)** GO enrichment analysis of the differentially expressed genes of this case, the top 30 biological processes (BP) term were listed.

Single-cell transcriptome sequencing analysis was performed on fresh tumor tissue obtained during two surgeries, 89 cells from the first surgery and 120 cells from the second surgery. First, a modified Single-cell Tagged Reverse Transcription sequencing (STRT-seq) technique (6) was applied and the single-cell RNA sequencing (scRNA-seq) data was obtained through Illumina sequencing. Then, information on chromosome copy number variation (CNV) was obtained by the InferCNV method based on scRNA-seq data, and a comparison of the CNV profiles of the two specimens revealed that two specimens had gain in chromosome 4, while chromosome 11 deletion was only detected in the specimen obtained from the first surgery, thus suggesting the possible existence of temporal and spatial heterogeneity within the tumor **(** **Figure 3A** **)**. Then, all the 209 cells were analyzed by principal component analysis (PCA), and the results showed that the cells were almost clustered together, and only little part of cells (7/209) had cell cycle heterogeneity, which indicated that the heterogeneity within the tumor was small and about 3% of cells was in the process of mitosis **(** **Figure 3B** **)**. At last, the differentially expressed genes of this case were analyzed and the biological processes (BP) corresponding to them were clarified by Gene Ontology (GO) enrichment analysis, in which the biological processes that were more involved were “Cytoskeleton Organization”, “anatomical structure development”, and “cellular component assembly involved in morphogenesis”**(** **Figure 3C** **)**.

## Discussion

One category of tumors highlighted in 2017 WHO classification is neuronal and paraneuronal tumors, which includes gangliocytoma and mixed gangliocytoma-adenoma, neurocytoma, paraganglioma and neuroblastoma, all of which are rare tumors, but they are of particular importance in the differential diagnosis of pituitary tumors (1, 2, 7, 8). This study highlights a case of mixed gangliocytoma-adenoma, which has a very low incidence, found to be only 0.29% (14/4891) in a retrospective German study that included 4891 pituitary lesions (3). Currently, there are three hypotheses regarding the pathogenesis of the disease: 1) Neuronal differentiation hypothesis. It is believed that most ganglion cell tumors in the sellar are the result of neuronal differentiation and chemosis in pituitary adenomas. Evidence supporting this hypothesis includes that both pituitary adenomas and ganglion cells can be observed microscopically (9). In a pathological study, Mikami et al. found that mixed gangliocytoma-adenoma is an intermediate morphological cell that lies between gangliocytoma and GH or PRL cell adenoma (10). It is generally believed that ganglion cells have no hormone-secreting capacity, but Li et al. found positive pituitary hormone expression in some tumor cells by performing neuron-related immunohistochemical staining of ganglion cell tumors in the sellar area (11). In addition, *in vitro* culture of anterior pituitary cells revealed that they can be converted into ganglion cells with different degrees of differentiation, providing a theoretical basis that pituitary adenomas can undergo neuronal transformation (12). 2) Residual ganglion cell hypothesis. It is believed that the ganglion cells that originally disappeared from the posterior pituitary gland due to excessive differentiation, like hypothalamic neurons, had the ability to release prohormone-releasing hormone, which can stimulate the occurrence of pituitary adenoma (13). At the same time, the ganglion cells are stimulated by the excessive secretion of prohormone-releasing hormone from the adenohypophysis and hypothalamic neurons, and eventually become tumorigenic (9). 3) Pituitary multipotent stem cell hypothesis (14). In 2006, Kontogeorgos et al. found that typical pituitary adenoma cells could express NFP, suggesting that pituitary adenomas have neuronal properties (15).

The clinical and imaging manifestations of mixed gangliocytoma-adenoma are essentially indistinguishable from pituitary adenoma, making preoperative diagnosis more difficult. This case presented clinically with acromegaly. On cranial MRI, the tumor was predominantly solid with occasional cystic changes. Also, it showed aggressive growth on imaging, invading the left cavernous sinus and encircling the internal carotid artery. So, a staged resection strategy was adopted. However, due to the tough texture of the cavernous sinus segment, a total dissection could not be achieved, and the oculomotor nerve was disturbed during the operation which leaded to postoperative oculomotor nerve palsy. In recent years, with the development of endoscopic technique, some medical centers had attempted to resecting the intrasellar and parasellar lesions through a combined endoscopic transsphenoidal and craniotomy strategy (16, 17). Three months after the postoperative interval, the patient underwent Gamma Knife treatment, but the results were not satisfactory. Gamma knife radiosurgery has emerged as a relatively safe management option for patients (e.g., when cavernous sinus or dural invasion prevents total resection) with adjuvant treatment after sub-total resection of the lesion, or as primary treatment for selected patients when the risk of surgery is considered too high. The reported incidence of new endocrine disorders after radiosurgery was typically in the range of 25% to 40%, with a lower incidence of hypopituitarism approaching 20% (18). With recent multicenter study series showing that functional pituitary adenomas require higher therapeutic doses than nonfunctional pituitary adenomas and that patients with high doses of pituitary stalk irradiation in Gamma Knife treatment are at higher risk for post-Gamma Knife treatment endocrine disorders, the risk burden of achieving long-term endocrine remission remains a concern in current treatment approaches (18, 19). Eventually, the patient died of sudden syncope due to secondary hyperglycemic hyperosmolar syndrome. It has been reported that 15% to 38% of patients with acromegaly develop reduced glucose tolerance or diabetes, and high GH leads to abnormal glucose metabolism through several pathways, with insulin resistance being the predominant mechanism (20). High GH, advanced age, long duration of disease, family history of diabetes, and hypertension are risk factors for the development of abnormal glucose tolerance in patients with acromegaly (21). Through the diagnosis and treatment process of this case, we further understood the refractory nature of mixed gangliocytoma-adenoma, especially the growth hormone type, which is often accompanied by systemic damage, and can greatly affect the patient’s quality and expectancy of life. The authors’ opinion was: 1) Surgical resection remains the preferred first-line treatment; after all, surgery can immediately reduce GH and IGF-1 to lower levels as well as improve signs and symptoms and obtain a pathologic histologic confirmation. 2) In surgery, resection of pituitary adenomas extending into the cavernous sinus is aggressively pursued; however, residual tumor in the cavernous sinus is inevitable. 3) The efficacy of Gamma knife treatment of residual or recurrent MGA presenting as acromegaly still needs to be tested. 4) It has also been shown that appropriate use of growth-inhibiting drugs during Gamma Knife treatment, while waiting for IGF-1 normalization, may allow patients with acromegaly to achieve endocrine remission. However, the cost of treatment with growth inhibitors is very high and imposes a heavy financial burden on patients. Therefore, it is important to explore effective lifelong alternative treatment options for patients with persistent acromegaly after surgical resection.

Currently, the diagnosis of MGA relies on postoperative pathological examination, including histomorphological observation and immunohistochemical staining (4, 22). Ganglion cells are distributed in clusters or scattered in the rich nerve fiber network, with eosinophilic cytoplasm and binucleated or multinucleated; while pituitary adenoma cells are diffusely distributed, with round or ovoid nuclei, uniformly slender and loose chromatin, granular cytoplasm, partially vacuolated cytoplasm, and abundant interstitial blood sinuses. Often, these neuronal tumors are situated cap-like over the adenomas, with no obvious boundary between them. Immunohistochemical staining reveals positive expression of markers of ganglicytoma (e.g. SYN, Ck8/18, CgA, S-100, etc.) and positive expression of pituitary cell hormones (e.g. GH, PRL, TSH, ACTH, LH, FSH, etc.). It has also been reported that GHRH is expressed in ganglion cells in MGA with acromegaly (23), and CRH is expressed in ganglion cells in MGA with Cushing’s syndrome (24). As for adenoma component in MGA, half is GH adenoma type as reported by Saeger in a Germany cohort with 14 cases included, but there are also reports related to prolactin adenoma, gonadotropin, and ACTH adenoma (3, 25, 26). In this case, tissue observation, immunohistochemistry, and electron microscopic ultrastructural observation were performed and were consistent with previous reports.

In addition, to explore the intratumoral heterogeneity of MGA, we also performed a pioneering single-cell transcriptome sequencing analysis of specimens from both surgeries to explore the tumor cell composition structure, intratumoral heterogeneity of the tumor, and functional types of cells at the single-cell level. Single-cell transcriptome sequencing is a relatively new technology for high-throughput transcriptome sequencing at the individual cell level, which can effectively address the challenges of cell heterogeneity and transcriptome heterogeneity within cell populations that are masked by bulk RNA-seq, and help discover new rare cell types and gain insight into the mechanisms of expression regulation during cell growth (27, 28). Through single-cell level studies, we found some temporal and spatial heterogeneity in the intratumoral cells of MGA, as evidenced by differences in the CNV profiles of the two specimens. However, at the transcriptomic level, the transcriptomic gene expression patterns of the cells obtained from the two specimens were extremely similar, with only a small fraction of cells in mitosis, which in turn suggested that MGA is a tumor with less intratumoral heterogeneity and inactive proliferation, which is consistent with previous studies of pituitary adenomas at the bulk level (29–31). We then focused on the functional properties of the adenomatous component of growth hormone in MGA, and obtained the more active biological processes within the tumor by GO analysis. However, unfortunately, no ganglion cells were found in the 209 single cells we obtained by analyzing their gene signature. We speculated that this might be related to the insufficient number of cells taken, therefore, we also intended to further search for ganglicytoma cells by increasing the specimen volume in future studies.

## Conclusion

Mixed gangliocytoma-adenoma presenting as acromegaly is a rare CNS neoplasm which is not that benign, because it’s growth hormone over-secretion behavior can cause severe damage to various systems of the body, and once it shows an invasive growth pattern, it is hard to achieve anatomic and functional resection, thus seriously affects the quality and span of patients’ life. MRI findings are useful to make a surgery planning, but the histopathological examination, including a full immunohistochemistry panel, is essential to correctly diagnose MGA. With a high probability, single-cell molecular testing can help deeper understanding the pathogenesis of the disease, but more cases will be needed in the future.

## Data availability statement

The datasets presented in this study can be found in online repositories. The names of the repository/repositories and accession number(s) can be found in the article/**Supplementary Material**.

## Ethics statement

Written informed consent was obtained from the minor(s)’ legal guardian/next of kin for the publication of any potentially identifiable images or data included in this article.

## Author contributions

CL wrote the manuscript. DZ and DF reviewed and edited the manuscript. All authors contributed to the article and approved the submitted version.

## Supplementary material

Supplementary Figure 1 The timeline of the clinical course of the case.
