# Supplementary figures and images for: Case report: Clinical and single-cell transcriptome sequencing analysis of a mixed gangliocytoma-adenoma presenting as acromegaly
Source: Front Oncol. 2022 Dec 8;12:1088803. doi: 10.3389/fonc.2022.1088803 (PMC9772982; doi:10.3389/fonc.2022.1088803)

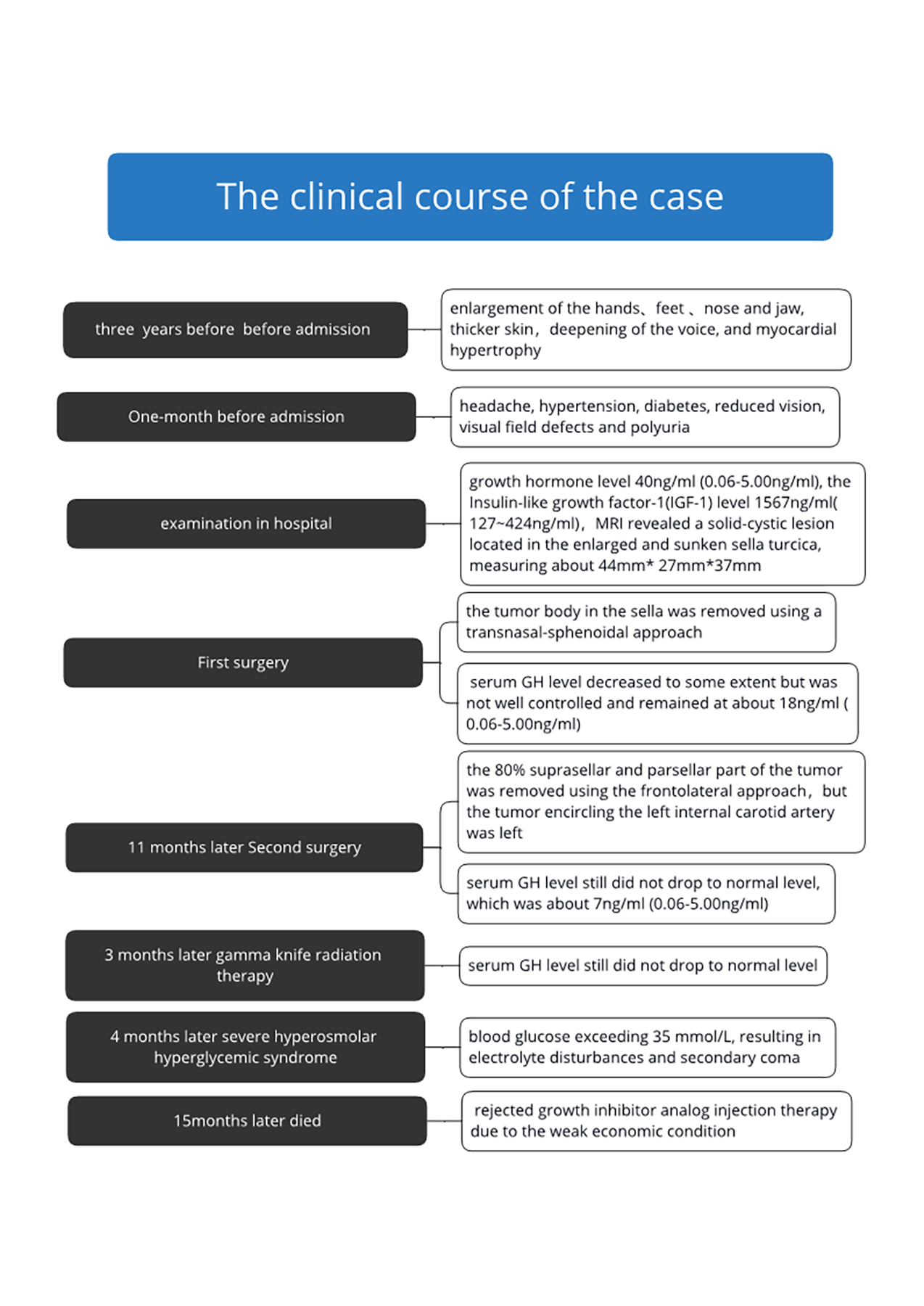

Supplement: Supplementary Figure 1 — The timeline of the clinical course of the case. [file Image_1.tif]
